# An amino acid-based supramolecular nanozyme by coordination self-assembly for cascade catalysis and enhanced chemodynamic therapy towards biomedical applications†

**DOI:** 10.1039/d1na00619c

**Published:** 2021-09-16

**Authors:** Enhui Song, Yongxin Li, Lili Chen, Xiaopeng Lan, Changshun Hou, Chunlei Liu, Chunzhao Liu

**Affiliations:** State Key Laboratory of Bio-fibers and Eco-Textiles, Institute of Biochemical Engineering, Affiliated Qingdao Central Hospital, College of Materials Science and Engineering, Qingdao University Qingdao 266071 China czliu@qdu.edu.cn liyx@qdu.edu.cn alanhenry14@126.com; Department of Biomedical Sciences, City University of Hong Kong Hong Kong 999077 P. R. China changshou2-c@my.cityu.edu.hk

## Abstract

The clinical translation of chemodynamic therapy has been highly obstructed by the insufficient intracellular H_2_O_2_ level in diseased tissues. Herein, we developed a supramolecular nanozyme through a facile one-step cooperative coordination self-assembly of an amphipathic amino acid and glucose oxidase (GOx) in the presence of Fe^2+^. The results demonstrated that the supramolecular nanozyme possessed cascade enzymatic activity (*i.e.*, GOx and peroxidase), which could amplify the killing efficacy of hydroxyl radicals (˙OH) *via* self-supplying H_2_O_2_, finally achieving synergistic starvation–chemodynamic cancer therapy *in vitro*. Additionally, this cascade nanozyme also exhibited highly effective antibacterial activity on *Escherichia coli* (*E. coli*) and *Staphylococcus aureus* (*S. aureus*) without the need for additional H_2_O_2_. This work provided a promising strategy for the design and development of nanozymes for future biomedical applications.

## Abstract 2

We developed a supramolecular nanozyme through the strategy of multicomponent coordination self-assembly using a bio-derived amino acid, glucose oxidase and Fe^2+^ as building blocks, which exhibited great potential towards biomedical applications.

## Graphical abstract

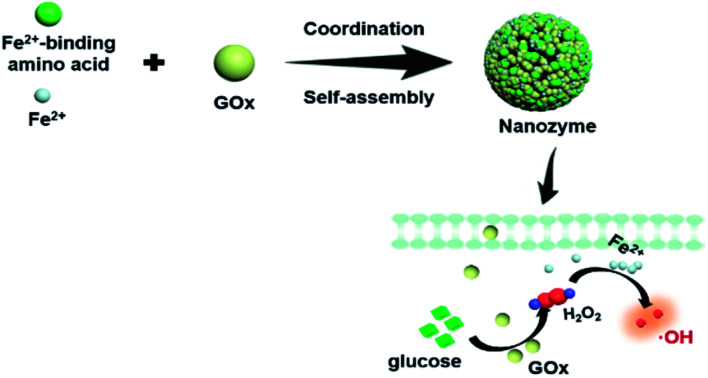

## Introduction

Chemodynamic therapy (CDT), which can convert less reactive hydrogen peroxide (H_2_O_2_) into the most harmful hydroxyl radical (˙OH) through the metal catalyst (*e.g.*, Fe^2+^, Mn^2+^, and Cu^+^) -mediated Fenton reaction or Fenton-like reaction,^1–5^ is considered to be a promising novel modality for relevant diseases (*e.g.*, cancer and pathogenic bacterial infection) because of its local selectivity and negligible side effects.^6,7^ However, the therapeutic outcomes of CDT have been highly limited due to the insufficiencies of endogenous H_2_O_2_,^8–10^ which significantly compromises the antitumor or antimicrobial effects. Therefore, the incorporation of a H_2_O_2_-supplemented functionality into conventional CDT strategies has been exploited for potentiating their therapeutic efficiencies.^11,12^

Glucose oxidase (GOx) has attracted particular interest as a natural H_2_O_2_-generating enzyme, which can catalyze the oxidation of glucose into gluconic acid and H_2_O_2_.^13^ Therefore, a variety of platforms have been developed so far to combine GOx-based starvation therapy with CDT for cancer treatment. For instance, Qing^14^ and co-workers developed a MnO_2_ nanosheet-based nanoreactor for co-enhanced chemodynamic and starvation therapy against tumor hypoxia, and the GOx and fluorescent reporters (FRs) were co-assembled on MnO_2_ nanosheets, which were enwrapped with hyaluronic acid for realizing tumor targeting. In another study by Ke^15^ and co-workers, the nanoreactors were constructed from polyprodrug polymersomes incorporating ultrasmall iron oxide nanoparticles and GOx to activate cascade reactions for orchestrated cooperative cancer treatment. Nevertheless, these methods either suffer from safety concerns due to the premature leakage of payloads and long-term toxicity of the delivery vehicles, or are restricted by insufficient drug loading, uncontrollable drug ratios and complicated synthetic routes.^16–18^ Therefore, it is extremely urgent but challenging to construct a simple, safe and efficient nanoplatform integrating GOx and CDT catalysts with high loading efficiency for synergistic starvation–chemodynamic therapy.

Nanozymes have aroused increasing interest in recent years due to their superior stability, low cost, and tunable catalytic activities compared with natural enzymes.^19–21^ For instance, Shi *et al.*^22^ reported the construction of an efficient dual inorganic nanozyme-based nanoplatform, which exhibits cascade enzymatic activity (*i.e.*, GOx and peroxidase) within the tumor microenvironment based on ultrasmall Au and Fe_3_O_4_ NP coloaded dendritic mesoporous silica NPs, whereas the long-term toxicity of inorganic materials significantly hindered its clinical translation. As an alternative strategy, simple biomolecules of biological origin in the design of nanomaterials are vigorously pursued,^23–25^ but designing and engineering a pluralistic nanozyme starting from small molecule combinations and their cooperative interactions still remain in the infant stage.

**Fig. 1:**
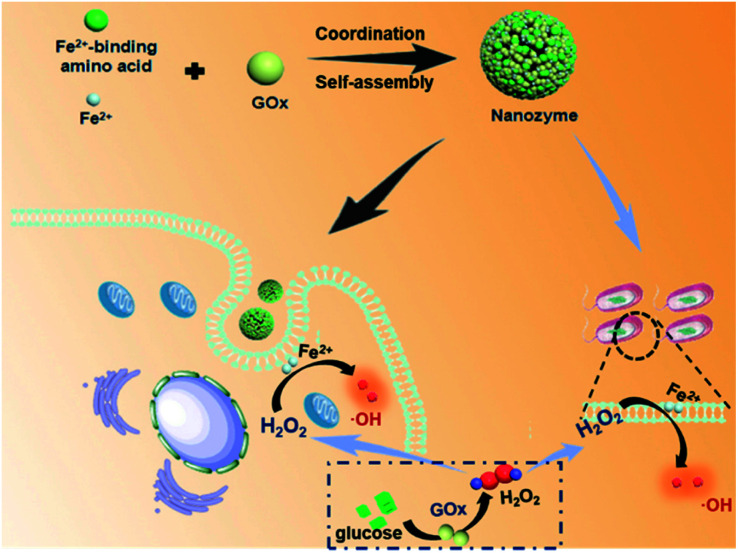
Schematic illustration of ferrous ion-driven coordination self-assembly of the supramolecular nanozyme using GOx and amino acids as metal-binding motifs, as well as its mechanistic actions for anticancer and antibacterial therapy.

In nature, metal-ion binding plays a key role in regulating the supramolecular nanoarchitectonics and catalytic activity of metalloenzymes (*e.g.*, peroxidase and catalase).^26,27^ In light of this, coordination-driven self-assembly could be regarded as a versatile strategy to fabricate biomimetic nanozymes through the interactions of the metal ions with metal-binding amino acids.^28–30^ And amino acids have shown flexibility and versatility in designing self-assembled materials due to the inherent advantages such as molecular simplicity, stability, low immunogenicity and easy availability.^31–33^ Moreover, GOx is also composed of multiple amino acids. Herein, we constructed a supramolecular nanozyme using the strategy of amino acid coordination driven self-assembly. By using metal-binding amino acids, GOx, and ferrous ions as building blocks, well-defined, uniform supramolecular nanozymes were obtained on the basis of a combination of coordination and multiple noncovalent interactions (Fig. 1). After entering into tumor cells, the nanozyme released the GOx first, which could decompose glucose into gluconic acid and H_2_O_2_, cutting off the nutrient supply to induce starvation therapy. Then, the generated H_2_O_2_, as well as the endogenous H_2_O_2_ in tumor cells, could activate and improve the peroxidase activity of the nanozyme for cascade catalytic generation of highly toxic ˙OH *via* the Fenton reaction, leading to enhanced CDT performance. What is more is that this cascade nanozyme also exhibited excellent antibacterial performance toward both *E. coli* and *S. aureus*, holding great promise for future biomedical applications.

## Experimental

### Preparation of Fmoc-L/Fe nanoparticles and Fmoc-L/Fe/GOx nanoparticles

A stock solution of Fmoc-Leu (Fmoc-L) was prepared by dissolving Fmoc-L in DMSO to give a concentration of 100 mM. Ferrous sulfate (FeSO_4_) was dissolved in distilled water to form 10 mM stock solution. Afterwards, Fmoc-L stock solution (10 μL) and FeSO_4_ stock solution (100 μL) were added to distilled water (890 μL), and a small amount of Tris (1 M) solution was added to adjust the final pH of the system to neutral. The turbidity indicated the formation of Fmoc-L/Fe nanostructures. The preparation method of Fmoc-L/Fe/GOx nanoparticles is similar to that of Fmoc-L/Fe nanoparticles: 100 μL FeSO_4_ stock solution and 10 μL GOx (10 mg mL^−1^) were mixed, and then diluted with distilled water (880 μL), followed by the addition of 10 μL Fmoc-L stock solution. Then a small amount of Tris solution was added to adjust the final pH of the system to neutral, the colloidal suspension indicating the formation of nanoparticles.

### Quantitative analysis of Fmoc-L/Fe nanoparticles

The solutions of Fmoc-L/Fe nanoparticles were centrifuged at 14 000 rpm for 10 min, and the residues were re-suspended in distilled water. This process was repeated three times to remove any free Fe^2+^ not involved in the nanoparticles. These nanoparticles were then re-dispersed in distilled water (200 μL). After the addition of DMSO (100 μL) into the samples, the content of Fmoc-L was determined by measuring the UV-vis absorption spectra and comparing with the pre-established calibration curves. For the measurement of Fe^2+^, the nanoparticles were mixed with aqua regia (a mixture of nitric acid and hydrochloric acid) and measured by inductively coupled plasma-optical emission spectroscopy (ICP-OES, PerkinElmer Avio 200).

### Determination of the GOx content involved in Fmoc -L/Fe/GOx nanoparticles

GOx was labeled first with fluorescein isothiocyanate (FITC), and Fmoc-L/Fe/FITC–GOx nanoparticles were prepared. Then the nanoparticles were centrifuged at 14 000 rpm for 10 min, and the supernatant was taken to measure the absorption at 490 nm. The encapsulation efficiency (EE) was calculated according to the equation: EE (%) = (total FITC–GOx − free FITC–GOx)/total FITC–GOx × 100%. Data are expressed as EE% ± standard deviation (SD) of three independent experiments.

### ˙OH production

The solutions of 10 μg mL^−1^ methylene blue (MB), 50 mM H_2_O_2_, and 2 mM Fmoc-L/Fe nanoparticles were mixed, and the degradation of ˙OH-induced MB was detected by observing the change in absorbance at 660 nm compared with that of the control group. For terephthalic acid (TPA) oxidation, 5 mM TPA solution was prepared in 2 mM NaOH solution, then 10 mM Fmoc-L/Fe nanoparticles and 50 mM H_2_O_2_ were added, and after a few minutes, the fluorescence intensity at ∼440 nm under 312 nm wavelength excitation was measured.

### Catalytic activity assessment of GOx

The GOx activity was estimated by the assessment of the product, gluconic acid, which causes the pH to drop. In detail, 8 mM Fmoc-L/Fe/GOx nanoparticles ([GOx] = 0.8 mg mL^−1^) and 10 mg mL^−1^ glucose were mixed at 37 °C and the initial pH of the solution was adjusted to pH 6.0. The pH of the solution was measured with a pH meter over time.

### Cell line and culture conditions

Human breast cancer cells MCF-7 cells were maintained in RPMI-1640 medium supplemented with fetal bovine serum (10%), and penicillin–streptomycin (100 units per mL and 100 μg mL^−1^, respectively). The cells were grown at 37 °C in a humidified 5% CO_2_ atmosphere.

### Cellular uptake of Fmoc-L/Fe/GOx nanozymes

The MCF-7 cells were seeded onto Petri dishes (1 × 10^5^ cells per well) and incubated for 24 h at 37 °C. MCF-7 cells were treated with Fmoc-L/Fe/FITC–GOx nanoparticles ([Fe^2+^] = 200 μM, [GOx] = 1 μg mL^−1^) for 2 or 12 h. Finally, the cells were examined with a Zeiss laser scanning microscope. FITC–GOx was excited at 488 nm and its fluorescence was monitored at 493–600 nm.

### Intracellular ˙OH detection

The MCF-7 cells were seeded onto Petri dishes (1 × 10^5^ cells per well) and incubated for 24 h at 37 °C. MCF-7 cells were treated with Fmoc-L/Fe ([Fe^2+^] = 200 μM) nanoparticles + H_2_O_2_ (200 μM), H_2_O_2_, Fmoc-L/Fe nanoparticles, Fmoc-L/Fe/GOx nanoparticles ([GOx] = 1 μg mL^−1^) +glucose (1 mg mL^−1^), and Fmoc-L/Fe/GOx nanoparticles + glucose + H_2_O_2_ for 12 h, respectively. The medium was removed and the cells were further incubated with 2′,7′-dichlorodihydrofluorescein diacetate (DCFDA, Sigma–Aldrich, 30 μM) for 30 min. Finally, the cells were examined with a Zeiss laser scanning microscope. DCFDA was excited at 488 nm and its fluorescence was monitored at 493–600 nm. The images were digitized and analyzed using the Zen software.

### Cytotoxicity assay

Approximately 1 × 10^4^ MCF-7 cells per well in RPMI medium were inoculated in 96-well plates and incubated for 12 h. The cells were treated with different concentrations of Fmoc-L/Fe nanoparticles ([Fe^2+^] = 200, 100, 50, 25 and 12.5 μM) and Fmoc-L/Fe/GOx nanoparticles ([GOx] = 0.0625, 0.125, 0.25, 0.5, and 1 μg mL^−1^) for 12 h, under the conditions in the presence of H_2_O_2_ (200 μM) or not. The Fmoc-L/Fe/GOx nanoparticle group was also treated with glucose (1 mg mL^−1^). Finally, MTT assay was used to evaluate the cell viability.

### Bacterial culture and *in vitro* bacterial experiments

*E. coli* and *S. aureus* single colonies were transferred to Luria–Bertani (LB) medium and shaken at 160 rpm for 16 h at 37 °C. The bacteria were then diluted in saline to 1 × 10^9^ CFU mL^−1^. The bacterial growth inhibition was studied in LB medium: five groups of preparative bacterial suspensions were treated with Fmoc-L/Fe nanoparticles, H_2_O_2_, Fmoc-L/Fe nanoparticles + H_2_O_2_, Fmoc-L/Fe/GOx nanoparticles + glucose, and LB medium, respectively. The concentrations of Fe^2+^, H_2_O_2_, GOx and glucose were 1.5 mM, 100 mM, 800 μg mL^−1^ and 10 mg mL^−1^, respectively. After incubation at a speed of 300 rpm for 4 h at 37 °C, the bacterial concentrations were investigated by detecting the optical density at 600 nm (OD_600_). After the antibacterial growth inhibition evaluation, the bacteria of five groups were centrifuged and fixed with 4% formaldehyde for 30 min. Then the bacteria were treated with 30, 50, 70, 90 and 100% ethanol for 10 min in sequence. Finally, the morphology of bacteria after sputtering gold coating was observed by SEM.

## Results and discussion

### Self-assembly of Fmoc-L triggered by Fe^2+^

**Fig. 2:**
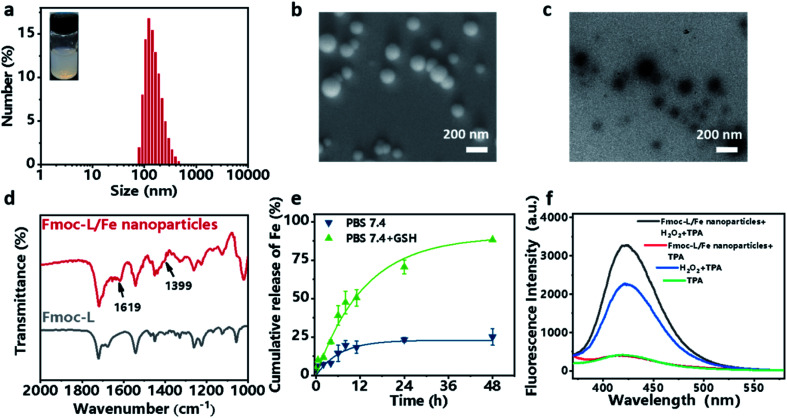
(a) DLS profile with a digital picture and (b) SEM image and (c) TEM image of the as-prepared Fmoc-L/Fe nanoparticles. (d) FTIR spectra of Fmoc-L/Fe and Fmoc-L. (e) GSH-responsive release profile of Fe^2+^ from Fmoc-L/Fe nanoparticles. (f) The change of fluorescence emission spectra of TPA after treatment with Fmoc-L/Fe nanoparticles with or without H_2_O_2_.

Fmoc-L (fluorenylmethoxycarbonyl-l-leucine) was chosen as the model amino acid owing to its inherent integration of a metal-binding essential amino acid, leucine, and a self-assembly moiety, the Fmoc group. Upon mixing a solution of Fmoc-L in dimethylsulfoxide (DMSO) and a solution of FeSO_4_ in distilled water, an opalescent and turbid colloidal suspension was obtained, indicating the formation of nanoparticles. The dynamic light scattering (DLS) profile (Fig. 2a) of the resulting suspension showed that Fmoc-L/Fe has narrow size distributions and an average diameter of 161.2 ± 68.01 nm. The scanning electron microscopy (SEM) image (Fig. 2b) and transmission electron microscopy (TEM) image (Fig. 2c) showed that Fmoc-L/Fe consists of spherical nanoparticles with sizes of approximately 160 nm, which is consistent with the DLS results. To investigate the self-assembly mechanism of the nanoparticle formation, the infrared (IR) spectrum of Fmoc-L/Fe was recorded and compared with that of Fmoc-L (Fig. 2d). Distinct bands at 1619 cm^−1^ and 1399 cm^−1^ in the spectrum of Fmoc-L/Fe corresponding to the asymmetric and symmetric stretching vibrations appeared, indicating that the carboxyl groups of Fmoc-L have been coordinated to the Fe ions.^34,35^ The quantitative component analysis further revealed that the molar ratio of Fmoc-L to Fe^2+^ was close to 6 : 1, which conforms to the stoichiometry of ferrous coordination mode. These results, in combination with our previous studies,^28^ suggested that the self-assembly process of Fmoc-L/Fe nanoparticles involved the formation of preliminary ferrous coordination complexes and further growth of the resulting complexes into advanced structures through multiple noncovalent interactions, such as hydrophobic interactions and π–π stacking of the aromatic motifs.

### Stimuli-responsive properties and peroxidase-like activity of Fmoc-L/Fe nanoparticles

Now that the formation of Fmoc-L/Fe nanoparticles is based on the synergy of coordination and other weak interactions, the complexes should be sensitive to environmental stimuli, such as overexpressed GSH in cancer cells, which could competitively bind to Fe^2+^. The release of Fe^2+^ from Fmoc-L/Fe nanoparticles was examined using phenanthroline, which can react with Fe^2+^ forming orange red complexes with a maximum absorption at 510 nm. As shown in Fig. 2e, Fmoc-L/Fe nanoparticles remained nearly stable in the absence of GSH, whereas the release efficiency of Fe^2+^ in the presence of 5 mM GSH significantly increased up to 75% during 48 h. These results indicated that Fmoc-L/Fe could be triggered by GSH to rapidly release Fe^2+^, which would act as a core reagent to initiate the Fenton reaction for generating ˙OH. Next, we evaluated the production of ˙OH from Fmoc-L/Fe nanoparticles using terephthalic acid (TPA) as a probe, which can be transformed into fluorescent 2-hydroxyterephthalic acid with a characteristic peak at 426 nm in the presence of ˙OH. As shown in Fig. 2f, the fluorescence intensity of Fmoc-L/Fe nanoparticles with the H_2_O_2_ treated-group increased significantly compared with that of Fmoc-L/Fe nanoparticles or the H_2_O_2_ alone group. In addition, methylene blue (MB) can be degraded quickly when H_2_O_2_ was added to Fmoc-L/Fe nanoparticles (Fig. S1†), whereas the degradation rate of MB by only H_2_O_2_ or Fmoc-L/Fe nanoparticles was relatively low. These results suggested the peroxidase-like activity of Fmoc-L/Fe nanoparticles to generate ˙OH.

### Preparation and characterization of the Fmoc-L/Fe/GOx cascade system

**Fig. 3:**
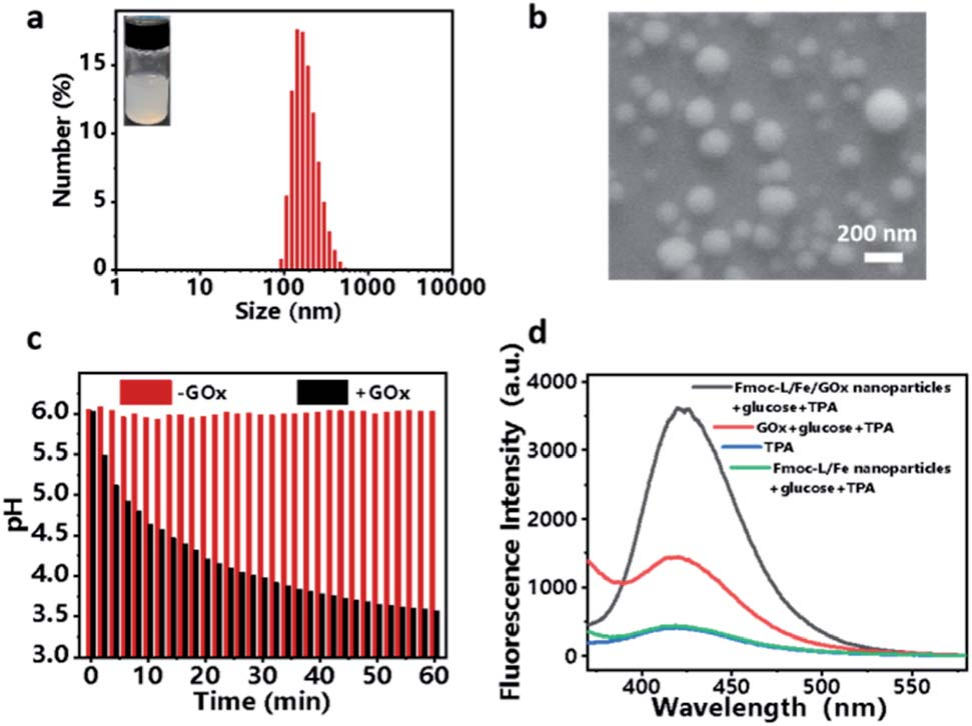
(a) DLS profile with a digital picture. (b) SEM image of Fmoc-L/Fe/GOx nanoparticles. (c) pH value changes of Fmoc-L/Fe/GOx and Fmoc-L/Fe solutions in the presence of glucose. (d) The change of fluorescence emission spectra of TPA after treatment with Fmoc-L/Fe/GOx or Fmoc-L/Fe in the presence of glucose.

Encouraged by the coordination self-assembly of Fmoc-L with Fe^2+^ and Fe^2+^-mediated robust peroxidase-like ability, we next studied the feasibility of the construction of a cascade nanozyme system with consideration of GOx. Multicomponent, cooperative coordination of Fmoc-L and GOx in the presence of Fe^2+^ in a one-step self-assembly process induced a colloidal suspension with an average size of 187.1 ± 69.54 nm for Fmoc-L/Fe/GOx (Fig. 3a). The SEM (Fig. 3b) and TEM images (Fig. S2†) further confirmed its spherical shape and size distribution, and indicated that the GOx incorporation has negligible influence on the morphology of Fmoc-L/Fe. The encapsulation efficiency of GOx in Fmoc-L/Fe/GOx was determined to exceed 99% by fluorescence measurements using fluorescein isothiocyanate (FITC)-labeled GOx. This high encapsulation efficiency was probably the result of cooperative coordination of GOx with Fmoc-L to Fe^2+^. The DLS results also revealed that the zeta potential value of Fmoc-L/Fe/GOx nanoparticles was −27.1 ± 4.50 mV, which was more negative than that of Fmoc-L/Fe nanoparticles, *i.e.*, −6.54 ± 2.52 mV. After validating the successful preparation, the cascade catalytic reaction of Fmoc-L/Fe/GOx was then detected. First, the catalytic capabilities of GOx were evaluated based on gluconic acid generation. The pH values were measured to indicate gluconic acid generation. As expected, a significant pH decline from 6.0 to 3.57 occurred after treatment with Fmoc-L/Fe/GOx while the pH of the solution remained stable in the absence of GOx (Fig. 3c), indicating the primary GOx activity of Fmoc-L/Fe/GOx. Subsequently, the peroxide-like activity of Fmoc-L/Fe/GOx was studied. As shown in Fig. 3d, Fmoc-L/Fe/GOx catalyzed the hydroxylation of TPA and caused the enhanced fluorescence intensity in the presence of glucose, and required no additional H_2_O_2_. The absorption of MB also exhibited a significant decrease after the addition of Fmoc-L/Fe/GOx nanoparticles and glucose (Fig. S3†). By contrast, the fluorescence intensity of TPA and the absorption of MB in solution treated with Fmoc-L/Fe nanoparticles showed no change. The effect of nanoparticles or free GOx alone on MB degradation is also very negligible. These observations obviously result from the H_2_O_2_ generation in the process of glucose being catalyzed by GOx and the following ˙OH generation catalyzed by Fe^2+^ using H_2_O_2_ as the substrate through the Fenton reaction. Thus, a pluralistic nanozyme platform with cascade enzymatic activities was achieved by incorporating natural GOx into peroxidase-mimicking Fmoc-L/Fe.

For biomedical applications, the high stability of nanoparticles is one of the necessary requirements. The stabilities of Fmoc-L/Fe nanoparticles and Fmoc-L/Fe/GOx nanoparticles were investigated first through creating 10-fold (v/v) dilutions in pure water and phosphate buffer (PBS) (pH 7.4). As shown in Fig. S5,† both Fmoc-L/Fe nanoparticles and Fmoc-L/Fe/GOx nanoparticles are highly stable as their size and size distributions have no discernable change. In addition, the stabilities of these nanoparticles were also evaluated in DMEM containing 10% FBS at 37 °C to mimic the physiological environment. The DLS results exhibited that the size of Fmoc-L/Fe nanoparticles and Fmoc-L/Fe/GOx nanoparticles remained unaltered (Fig. S5†). The high stability of these nanoparticles under different conditions indicated their great promise in biomedical applications.

### Intracellular ROS generation and cytotoxicity of Fmoc-L/Fe/GOx

**Fig. 4:**
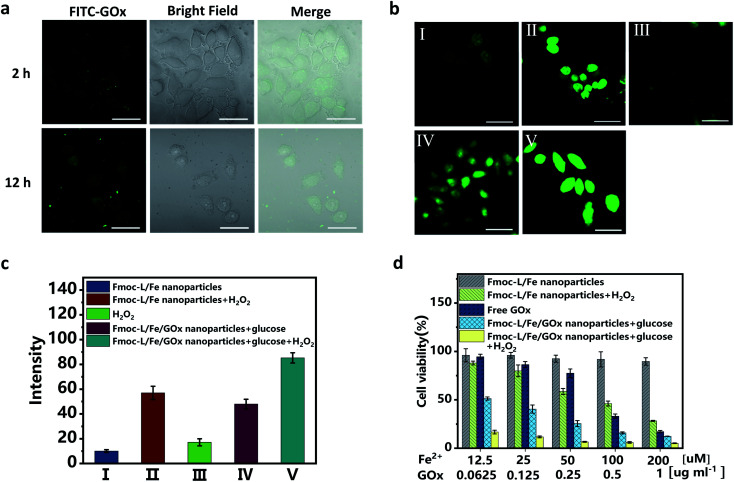
(a) CLSM images of MCF-7 cells incubated with the Fmoc-L/Fe/FITC–GOx nanoparticles showing intracellular uptake at 2 or 12 h. (b) CLSM images of MCF-7 cells after exposure to (I)–(V) to evaluate ˙OH production based on DCF-DA fluorescence intensity. (I) Fmoc-L/Fe, (II) Fmoc-L/Fe + H_2_O_2_, (III) H_2_O_2_, (IV) Fmoc-L/Fe/GOx + glucose, and (V) Fmoc-L/Fe/GOx + glucose + H_2_O_2_. All scale bars are 100 μm. (c) Quantified fluorescence intensity analysis for generated ˙OH according to (b). (d) MTT results of the viability of MCF-7 cells under different treatment conditions.

Since excellent cascade enzymatic activity and high stability of Fmoc-L/Fe/GOx were verified, *in vitro* catalytic cytotoxicity was further evaluated. The cellular uptake was first examined after incubating MCF-7 cells with the Fmoc-L/Fe/FITC–GOx nanoparticles. The confocal laser scanning microscopy (CLSM) images (Fig. 4a) show that the FITC–GOx fluorescence is mainly aggregated on the membranes at 2 h and entered gradually into the cytoplasm with the increase in incubation time, presumably by endocytosis.^36^ Next, the feasibility of cascade catalytic reactions occurring in living cells was studied based on ˙OH detection using dichlorofluorescein diacetate (DCF-DA) as an indicator. As shown in the CLSM images (Fig. 4b), only weak green fluorescence in cells was observed after treating with Fmoc-L/Fe nanoparticles or H_2_O_2_ alone. After incubation with Fmoc-L/Fe/GOx nanoparticles, the cells exhibited brighter green fluorescence, and the intensity was comparable with that of Fmoc-L/Fe nanoparticles with additional H_2_O_2_, suggesting that glucose decomposition by GOx caused the elevated H_2_O_2_ level, as well as the subsequent enhanced ˙OH generation catalysed by Fmoc-L/Fe in cells. So, when the cells were incubated with Fmoc-L/Fe/GOx nanoparticles plus H_2_O_2_, the fluorescence intensity became strongest. These observations were further reflected clearly by the quantified fluorescence intensity summarized in Fig. 4c. In one word, these results confirmed that the cascade catalytic activity of Fmoc-L/Fe/GOx nanoparticles could be realized in cancer cells to decompose glucose and generate higher amounts of ˙OH, which would achieve the combination of chemodynamic therapy and starvation therapy.

The *in vitro* therapeutic efficacy of Fmoc-L/Fe/GOx nanoparticles on MCF-7 cells was then examined through 3-(4,5-dimethylthiazol-2-yl)-2,5-diphenyltetrazolium bromide (MTT) assays. As shown in Fig. 4d, Fmoc-L/Fe nanoparticles exhibited limited cytotoxicity with a cell viability of about 90%, and upon addition of H_2_O_2_, the cell viability decreases to below 50% due to H_2_O_2_-mediated chemodynamic therapy because H_2_O_2_ itself is not cytotoxic at this concentration (Fig. S4†). Since GOx can consume glucose and cut off the supply of energy and nutrients to cancer cells to perform starvation therapy, free GOx showed apparent cytotoxicity to MCF-7 cells. Notably, Fmoc-L/Fe/GOx nanoparticles in the presence of H_2_O_2_ exhibited the strongest cytotoxicity with a cell viability of about 10% as compared to that of monotherapy groups. More importantly, even in the absence of H_2_O_2_, the Fmoc-L/Fe/GOx nanoparticle-treated group still exhibited excellent killing efficacy, indicating the synergistic therapy of starvation and potential chemodynamic therapy.

### Antimicrobial activity evaluation

**Fig. 5:**
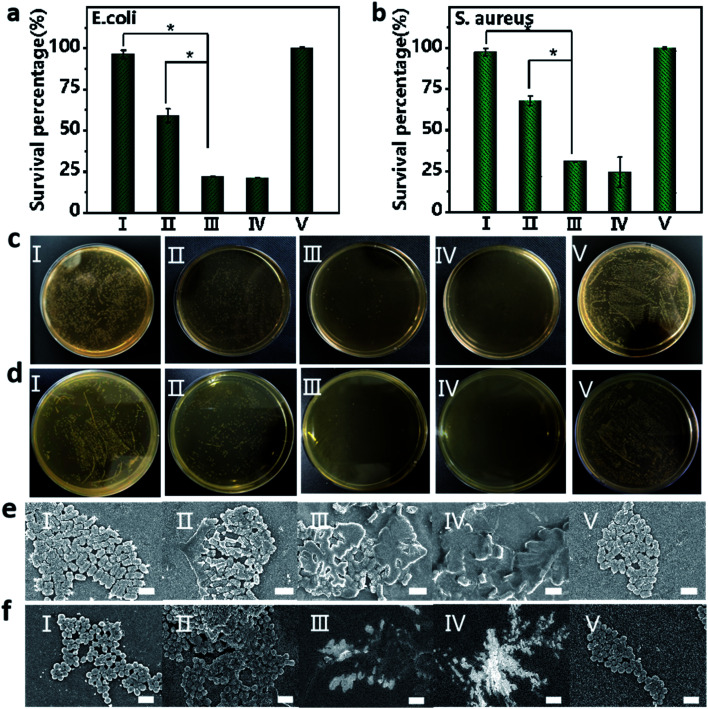
The bacterial viability of (a) *E. coli* and (b) *S. aureus* after treatment with (I)–(V), *P < 0.05 as calculated by Student's *t*-test. Photographs of bacterial colonies formed by (c) *E. coli* and (d) *S. aureus* after treatment with (I)–(V). SEM images of (e) *E. coli* and (f) *S. aureus* after exposure to (I)–(V). (I) Fmoc-L/Fe, (II) H_2_O_2_, (III) Fmoc-L/Fe + H_2_O_2_, (IV) Fmoc-L/Fe/GOx + glucose, and (V) blank control without any treatment. The scale bar is 2 μm.

Bacterial infection is a frequently encountered disease that severely threatens the health of human beings. Inspired by the excellent anticancer activity of the Fmoc-L/Fe/GOx nanozyme, we then evaluated its antibacterial activity in inhibiting the growth of Gram-negative *E. coli* and Gram-positive *S. aureus*. The excellent antibacterial activity of Fmoc-L/Fe was proved by the fact that the viabilities of *E. coli* and *S. aureus* were lower than 20% upon Fmoc-L/Fe nanoparticle treatment in the presence of H_2_O_2_ (Fig. 5a and b). As a control, the viabilities of *E. coli* and *S. aureus* were above 95% for Fmoc-L/Fe nanoparticles and 60% for H_2_O_2_-treated groups. Notably, the viabilities of *E. coli* and *S. aureus* were reduced to 20% in the Fmoc-L/Fe/GOx treated-group in the absence of H_2_O_2_, indicating the high catalytic efficiency of Fmoc-L/Fe/GOx in the cascade reaction of glucose to highly toxic ˙OH. Visible colonies of bacterial growth were further evaluated by antibacterial activity measurements (Fig. 5c and d). Almost no colonies were observed in Fmoc-L/Fe nanoparticles with H_2_O_2_ and Fmoc-L/Fe/GOx nanoparticle treated-groups, and the antibacterial activity results were consistent with those of the viability measurements. The morphology of bacteria was then characterized *via* SEM (Fig. 5e and f). The *E. coli* and *S. aureus* in Fmoc-L/Fe nanoparticle-treated groups presented a typical rod and spherical morphology with a smooth surface and intact cell walls, same as that of the blank group, demonstrating almost no toxicity toward *E. coli* and *S. aureus*. The H_2_O_2_ treated-group resulted in a few disruptions on the cell wall of the bacteria, indicating the slight cytotoxicity of H_2_O_2_. But the groups treated with Fmoc-L/Fe/GOx nanoparticles or co-incubated with Fmoc-L/Fe nanoparticles and H_2_O_2_ induced more serious damage to the bacterial cells, as reflected by the compressed morphology and rough surface. Thus, the prepared Fmoc-L/Fe/GOx nanoparticles possessed strong antibacterial activity with biocompatible glucose as the substrate, without the introduction of toxic H_2_O_2_.

## Conclusion

In conclusion, we fabricated a supramolecular nanozyme based on the multicomponent coordination self-assembly strategy using an amino acid, GOx and Fe^2+^ as building blocks for enhanced chemodynamic therapy towards relevant diseases. The resulting nanozyme exhibited a spherical shape, narrow size distribution, and impressive encapsulation efficiencies of hydrophilic GOx. The *in vitro* studies showed that this nanozyme could be internalized well by cancer cells and possessed strong cytotoxicity, which has also been proved from the combination of starvation and chemodynamic therapeutic effects. Importantly, Fmoc-L/Fe/GOx exhibited an excellent antibacterial efficiency without additional H_2_O_2_, which indicated the occurrence of cascade reactions from H_2_O_2_ generation by glucose oxidation to the production of highly active ˙OH *via* the Fenton reaction. Overall, this work provided a useful strategy *via* employing bio-derived molecules for preparing efficient nanozymes for future biomedical applications.

## Conflicts of interest

There are no conflicts of interest to declare.

## Supplementary Material

NA-003-D1NA00619C-s001
