# Supplementary material for: An amino acid-based supramolecular nanozyme by coordination self-assembly for cascade catalysis and enhanced chemodynamic therapy towards biomedical applications
Source: Nanoscale Adv. 2021 Sep 16;3(22):6482–9. doi: 10.1039/d1na00619c (PMC9417692; doi:10.1039/d1na00619c)
Supplement: NA-003-D1NA00619C-s001 [file NA-003-D1NA00619C-s001.pdf]

Electronic Supplementary Material (ESI) for Nanoscale Advances.
This journal is © The Royal Society of Chemistry 2021

Amino Acid-Based Supramolecular Nanozyme by Coordination Self-Assembly for Cascade Catalysis and Enhanced Chemodynamic Therapy towards Biomedical Applications

Enhui Song,‡[a] Yongxin Li,‡*[a] Lili Chen,[a] Xiaopeng Lan,*[a] Changshun Hou,*[b] Chunlei Liu,[a] and Chunzhao Liu*[a]

[a]State Key Laboratory of Bio-fibers and Eco-textiles, Institute of Biochemical Engineering, Affiliated Qingdao Central Hospital, College of Materials Science and Engineering, Qingdao University, Qingdao 266071, China.

[b]Department of biomedical science, City University of Hong Kong, Hong Kong 999077. P. R. China.

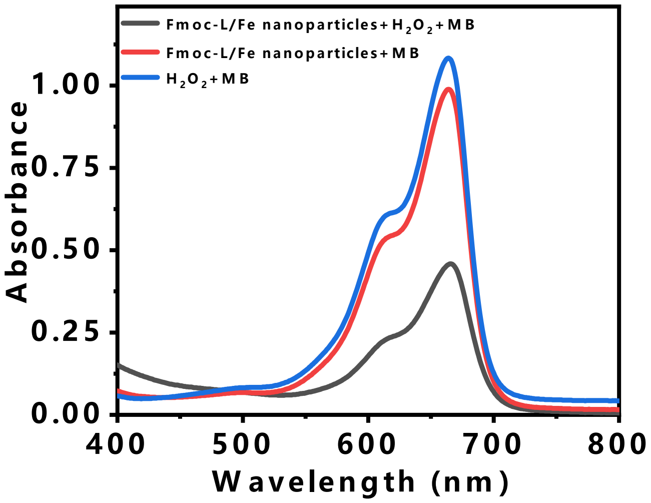

Fig. S1 The change on absorption of MB after treatment of Fmoc-L/Fe nanoparticles in the presence of H_2O_2 compared with nanoparticles or H_2O_2 alone.

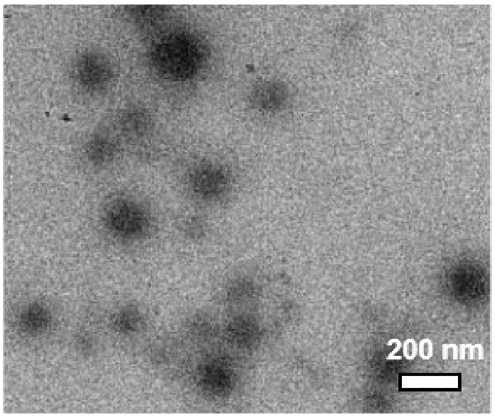

Fig. S2 TEM image of Fmoc-L/Fe/GOx nanoparticles.

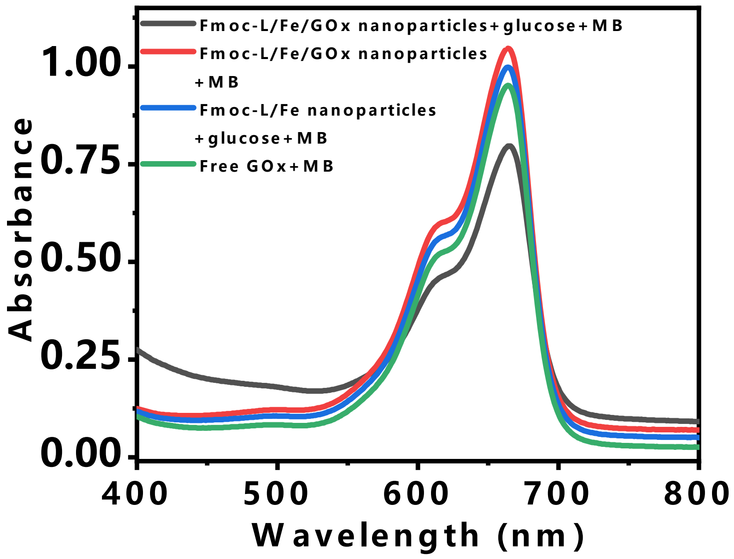

Fig. S3 The change on absorption of MB after treatment of Fmoc-L/Fe/GOx nanoparticles in the presence of glucose.

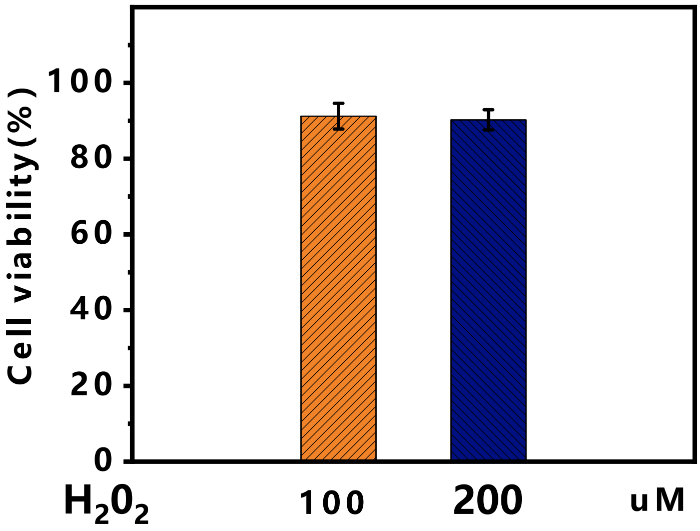

Fig. S4 The cell viability of MCF-7 cells after treatment with different concentration of H_2O_2.

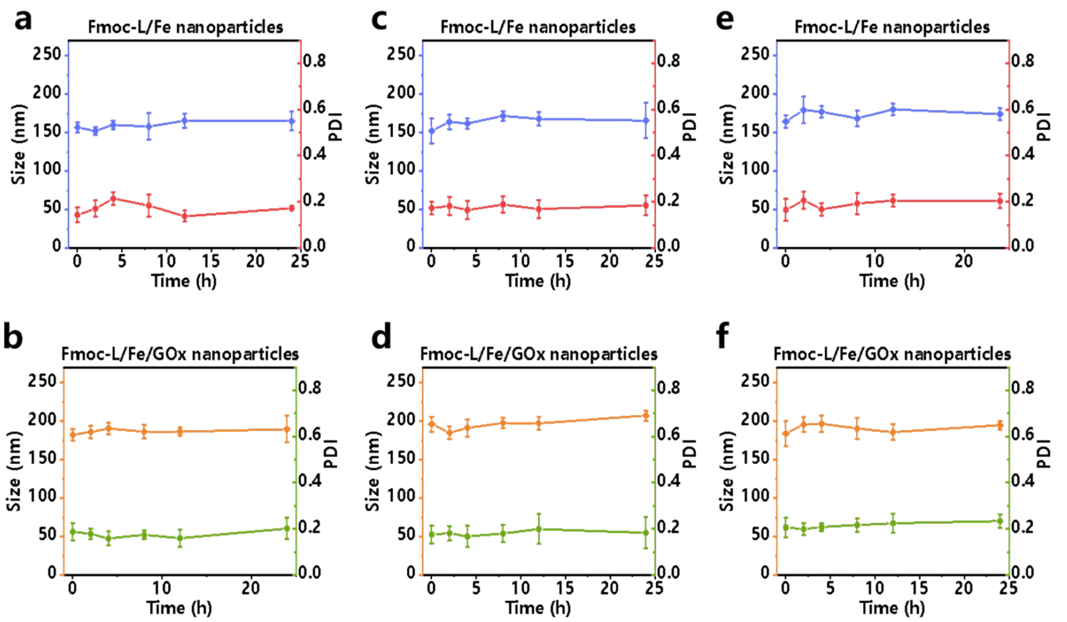

Fig. S5 The changes in the size and polymer dispersity index (PDI) of Fmoc-L/Fe nanoparticles (a) and Fmoc-L/Fe/GOx nanoparticles (b) after 10-fold (v/v) dilutions in water. The changes in the size and PDI of Fmoc-L/Fe nanoparticles (c) and Fmoc-L/Fe/GOx (d) nanoparticles in PBS. The changes in the size and PDI of Fmoc-L/Fe nanoparticles (e) and Fmoc-L/Fe/GOx nanoparticles (f) in DMEM with 10％ fetal bovine serum (FBS) at 37 ℃.
